# PReS-FINAL-2156: Analysis of gene expression and inflammation biomarkers in systemic juvenile idiopathic arthritis (SJIA) patients on canakinumab therapy

**DOI:** 10.1186/1546-0096-11-S2-P168

**Published:** 2013-12-05

**Authors:** N Nirmala, N Wulffraat, H Brunner, P Quartier, R Brik, L Mccann, H Ozdogan, L Rutkowska-Sak, R Schneider, V Gerloni, L Harel, M Terreri, K Houghton, R Joos, D Kingsbury, J Lopez-Benitez, S Radominski, A Brachat, S Bek, M Schumacher, M Valentin, H Gram, K Abrams, A Martini, N Ruperto, D Lovell

**Affiliations:** 1 NIBR, MA, USA; 2 PRINTO-Istituto Gaslini, Genova, Italy; 3 PRCSG, Cincinnati, OH, USA; 4 Necker-Enfant Malades Hospital, Paris, France; 5 Universidade Federal do Paraná, Curitiba, Brazil; 6 NIBR, Basel, Switzerland; 7 Novartis Pharmaceuticals Corp, NJ, USA

## Introduction

IL-1β, an inflammatory cytokine, plays an important role in SJIA, a rare autoinflammatory disease. Canakinumab (CAN), a selective fully human, anti-IL-1β monoclonal antibody, is reported to be efficacious in treating SJIA.

## Objectives

To characterize changes in peripheral blood gene expression and inflammatory proteins in SJIA patients (pts) treated with CAN and to identify baseline biomarkers that predict clinical response to CAN treatment.

## Methods

Levels of inflammatory biomarkers (IL6; total IL18) and gene expression profiles of active SJIA pts (aged 2-19 yrs) before and during CAN treatment enrolled in 2 phase III trials were analyzed.

## Results

### Gene expression

Transcriptional changes upon CAN treatment at Day 3 were assessed. When applying cut-offs of ≥2 fold and p ≤ 0.05, no transcript passed this filter for placebo pts and for CAN pts that were ACR30 (adapted pediatric ACR) non-responders at Day 15, while 171 probesets passed the filter for pts showing ≥ACR30 response. Pts who showed strong transcriptional changes also showed a strong ACR response (≥ACR50) at Day 15, while pts with <ACR50 at Day 15 showed a much weaker transcriptional response at Day 3. Strongly repressed genes included many known inflammation and innate immunity-related genes (eg, TLR1, TLR4, TLR5, TLR6, TLR8), including several members of the IL-1β signaling pathway, such as IL1β, IL1R1, IL1R2 and IL1RAP. A set of transcripts was identified for which high baseline expression levels predicted a subgroup of strong (≥ACR50) responders at Day 15. However, another subgroup of strong responders was indistinguishable from weak responders (≤ACR30) based on baseline transcript levels.

### Protein markers

IL-6 protein levels were strongly reduced by Day 3 (4.7× and 4.4× with p = 0.002 and 0.0001, for the 2 trials), and at Day 29 (12.5 × and 8.1 × with p = 0.01 and 0.00005,) while total IL18 levels remained largely unchanged until Day 29 and showed a moderate reduction only at Day 57. For IL6, stronger reduction at Day 3 and Day 29 was observed for pts who showed stronger ACR response at Day 15. Only 3 baseline samples were available from pts who developed macrophage activation syndrome during the studies.

## Conclusion

CAN treatment resulted in a rapid, strong reduction of many pro-inflammatory leukocyte transcripts and serum IL6. Compared with IL6, IL18 protein levels were reduced upon treatment much later and less strongly. About two thirds of pts with a strong treatment response (≥ACR50) were characterized by a set of leukocyte transcripts with high baseline levels and strong reduction upon CAN treatment. However, the remaining one third of CAN strong responders did not show these characteristic transcriptional patterns, suggesting some heterogeneity at the molecular level in SJIA pts showing strong response to CAN treatment.

## Disclosure of interest

N. Nirmala Shareholder of: Novartis, Employee of: Novartis, N. Wulffraat Grant/Research Support from: Roche, Abbott, Consultant for: Novartis, Pfizer, H. Brunner Consultant for: Novartis, Genentech, Medimmune, EMD Serono, AMS, Pfizer, UCB, Jannsen, Speakers Bureau: Genentech, P. Quartier Grant/Research Support from: Abbvie, Chugai-Roche, Novartis, Pfizer, Consultant for: Abbott/Abbvie, BMS, Chugai-Roche, Novartis, Pfizer, Servier, SOBI, Speakers Bureau: Chugai-Roche, Novartis, Pfizer, R. Brik Grant/Research Support from: Novartis, Consultant for: Novartis, L. Mccann: None declared., H. Ozdogan Grant/Research Support from: Novartis, L. Rutkowska-Sak: None declared., R. Schneider Consultant for: Hoffmann-La Roche, Novartis, Innomar Stragegies, V. Gerloni: None declared., L. Harel: None declared., M. Terreri: None declared., K. Houghton: None declared., R. Joos: None declared., D. Kingsbury: None declared., J. Lopez-Benitez: None declared., S. Radominski Grant/Research Support from: BMS, Novartis, Amgem, Pfizer, Sanofi, Janssen, Roche, Speakers Bureau: BMS, Pfizer, Sanofi, Janssen, GSK, A. Brachat Shareholder of: Novartis, Employee of: Novartis, S. Bek Employee of: Novartis, M. Schumacher Employee of: Novartis, M. Valentin Shareholder of: Novartis, Employee of: Novartis, H. Gram Employee of: Novartis, K. Abrams Shareholder of: Novartis, Employee of: Novartis, A. Martini Consultant for: To Gaslini Hospital: Abbott, Astrazeneca, BMS, Centocor Research & Development, Eli Lilly and Company, "Francesco Angelini", Glaxo Smith & Kline, Italfarmaco, Novartis, Pfizer Inc., Roche, Sanofi Aventis, Schwarz Biosciences gmbh, Xoma, Wyeth Pharmaceuticals Inc., Speakers Bureau: Bristol-Myers Squibb, Novartis, Astrazeneca, Glaxo Smith and Kline, N. Ruperto Grant/Research Support from: To Gaslini Hospital: Abbott, Astrazeneca, BMS, Centocor Research & Development, Eli Lilly and Company, "Francesco Angelini", Glaxo Smith & Kline, Italfarmaco, Novartis, Pfizer Inc., Roche, Sanofi Aventis, Schwarz Biosciences gmbh, Xoma, Wyeth Pharmaceuticals Inc., Speakers Bureau: Astrazeneca, Bristol Myers and Squibb, Janssen Biologics B.V., Roche, Wyeth/Pfizer, D. Lovell Grant/Research Support from: NIH, Consultant for: Astra-Zeneca, Centocor, Jansen, Wyeth, Amgen, Bristol-Meyers Squibb, Abbott, Pfizer, Regeneron, Hoffman La-Roche, Novartis, Genentech, Speakers Bureau: Genentech, Roche.

